# Pituitary macroadenomas: are combination antiplatelet and anticoagulant therapy contraindicated? A case report

**DOI:** 10.1186/1752-1947-1-74

**Published:** 2007-08-30

**Authors:** Tricia MM Tan, Carmela Caputo, Amrish Mehta, Emma CI Hatfield, Niamh M Martin, Karim Meeran

**Affiliations:** 1 Endocrine Unit, Hammersmith Hospitals NHS Trust, Imperial College Faculty of Medicine, London, UK; 2 Department of Radiology, Hammersmith Hospitals NHS Trust, Imperial College Faculty of Medicine, London, UK

## Abstract

**Background:**

Pituitary apoplexy is a life-threatening endocrine emergency that is caused by haemorrhage or infarction of the pituitary gland, commonly within a pituitary adenoma. Patients classically present with headache, ophthalmoplegia, visual field defects and altered mental state, but may present with a typical symptoms such as fever and altered conscious level.

**Case presentation:**

A 57-year-old female with a known pituitary macroadenoma was treated for suspected acute coronary syndrome with aspirin, clopidogrel and full dose enoxaparin. She developed a severe and sudden headache, nausea and vomiting and visual deterioration. A CT scan showed haemorrhage into the pituitary macroadenoma. She underwent neurosurgical decompression. Post-operatively her visual fields and acuity returned to baseline. She was continued on hydrocortisone and thyroxine replacement on discharge.

**Conclusion:**

This case illustrates the risks of anticoagulation in a patient with a known pituitary macroadenoma, and raises the issue of whether these tumours present a relative contraindication to the use of dual antiplatelet and anticoagulation in acute coronary syndrome.

## Background

Pituitary apoplexy is defined as haemorrhage or infarction of the pituitary gland. This occurs often in the context of a pituitary adenoma, although it can occur in normal pituitaries in patients with post partum haemorrhage (Sheehan's syndrome) [1]. This is an emergency because of the combination of secondary adrenal insufficiency, with compression of the optic chiasm and the III, IV, V and VI cranial nerves [2].

The prevalence of classical pituitary apoplexy in retrospective case series of patients undergoing pituitary surgery varies from 5% [3] to 9.1% [4]. In patients with non-functioning macroadenomas, who were not operated on and followed up for 85 +/- 13 months, 14% developed pituitary apoplexy [5]. The majority present with no previous history of pituitary adenoma, and their tumour is discovered when the apoplexy occurs [6]. Many precipitating factors have been described, from dopamine agonists [7], anticoagulation [8], head trauma [9], pituitary irradiation [10], to dynamic endocrine testing, most likely triggered by TRH administration [11].

## Case presentation

**Figure 1:**
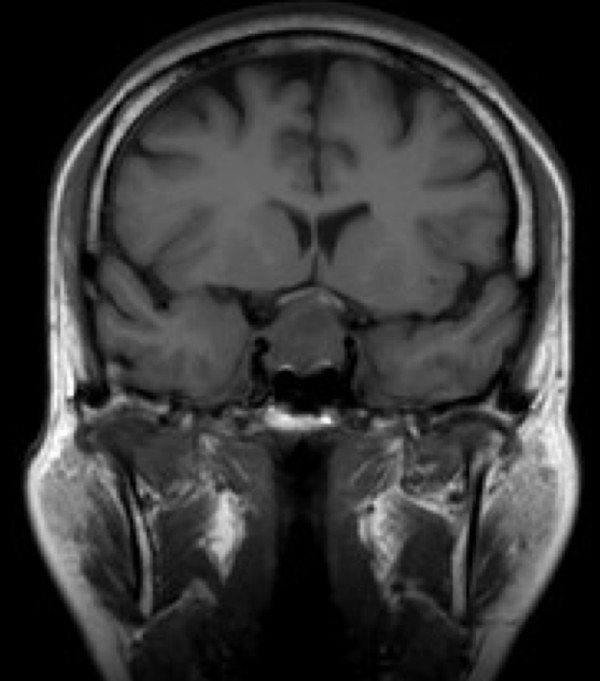
**MRI scan demonstrating pituitary macroadenoma**. A mixed cystic/solid mass is demonstrated lying within the pituitary fossa, bowing and indenting the optic chiasm.

A 57-year-old menopausal female, during evaluation for headache, was incidentally found to have a 20 × 16 mm mixed solid and cystic pituitary mass abutting the optic chiasm on MRI scanning (Figure 1). Endocrine testing showed that she had normal pituitary hormone levels with the exception of gonadotrophin deficiency. Formal perimetry initially showed full visual fields. Within 12 months, however, she developed a mild superior bi-temporal hemianopia. Despite this, she elected not to have surgery.

**Figure 2:**
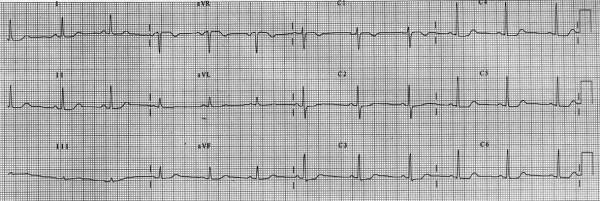
**ECG taken during apoplectic crisis**. Global ST depression is demonstrated, particularly in leads V2-V6. These changes were not present on her admission ECG and resolved after surgery.

Several months later, she was admitted to hospital with chest pain and treated for acute coronary syndrome with aspirin, clopidogrel and 1 mg/kg enoxaparin bd. She had no ECG changes suggestive of myocardial ischaemia or infarction. Her troponin level at 12 hours was undetectable. On the 2nd day of admission she developed a severe and sudden headache associated with nausea and vomiting. At this stage, her blood pressure was 144/85 mmHg and there were no neuro-ophthalmological symptoms or signs. The next day her symptoms continued and she noticed a constriction in her visual fields. She was febrile with an elevated C-reactive protein of 155 mg/l (normal < 5 mg/l). An ECG showed a new finding of global ST depression, but without chest pain (Figure 2).

**Figure 3:**
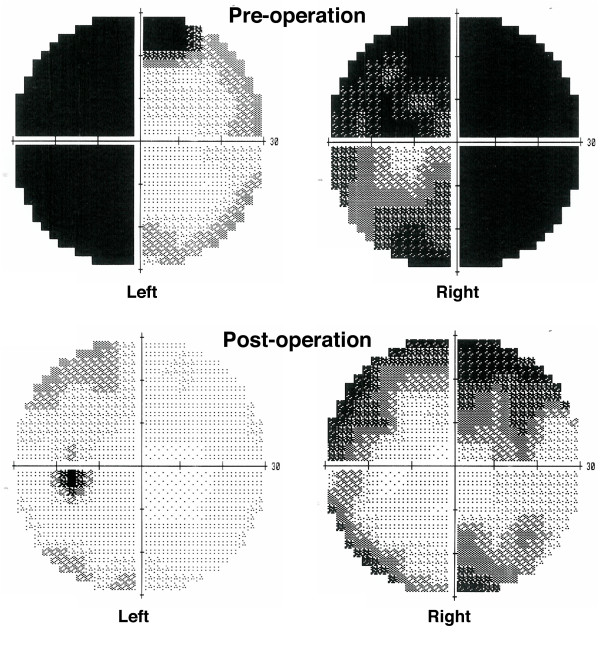
**Visual field tests before and after pituitary surgery**. The results of visual field testing are shown. The upper set, taken before the operation, demonstrate a temporal hemianopia in the left eye field and closure of three quadrants in the right eye field, sparing the inferior nasal quadrant. The lower set demonstrates the improved visual fields after surgery.

**Figure 4:**
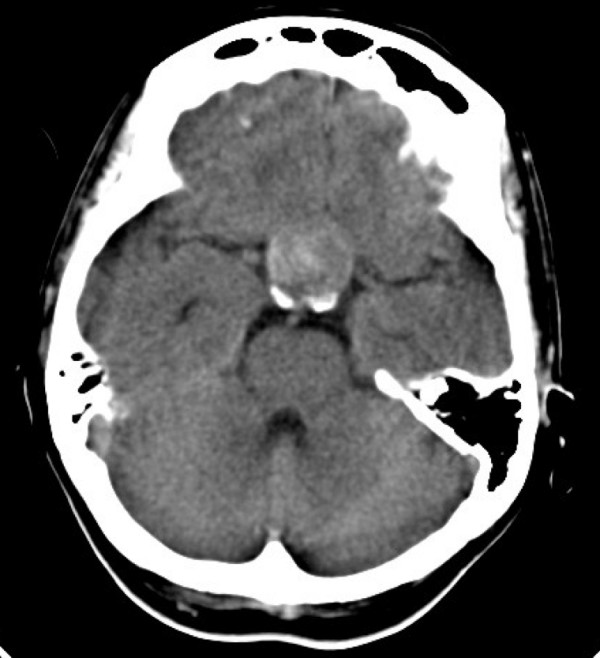
**CT scan of the pituitary demonstrating haemorrhage**. Patchy enhancement of the pituitary mass is seen indicating haemorrhage within the pituitary macroadenoma.

Perimetry confirmed deterioration in the patient's visual fields and acuities (Figure 3), and a CT scan showed haemorrhage into the pituitary macroadenoma (Figure 4). She was given intramuscular hydrocortisone, and underwent urgent trans-sphenoidal surgery and decompression of the apoplectic pituitary.

Post-operatively her visual fields and acuity returned to baseline (Figure 3). Her pyrexia ceased and CRP decreased. The ECG returned to normal. Cardiological investigations including exercise stress testing and a myocardial perfusion scan did not show evidence of ischaemic heart disease, implying that the global ST depression noted pre-operatively was the result of her pituitary apoplexy. She was discharged home on hydrocortisone and thyroxine replacement.

## Discussion

We describe a case of pituitary apoplexy in a patient who was already known to have a pituitary adenoma, and who was treated for acute coronary syndrome. With the modern treatment of acute coronary syndrome, an anticoagulation cocktail that includes aspirin, heparin and clopidogrel is employed. The addition of clopidogrel to aspirin and heparin has been demonstrated to reduce the incidence of further vascular events over aspirin and heparin alone. However, this is at the expense of a significant increase in rates of major bleeding (mainly gastrointestinal) from 2.7% to 3.7% [12]. The apoplectic crisis initially presented with fever, headache, nausea, and vomiting, and ST segment depression on ECG, a finding that has been reported with subarachnoid haemorrhage [13], but has not previously been reported in association with pituitary apoplexy. The risks of death or serious visual loss in the event of apoplexy are considerable, especially if there is diagnostic delay occasioned by a non-classical presentation, e.g. with fever of unknown origin, hyponatraemia and altered consciousness [6]. This situation can be compounded by non-diagnostic investigations such as non-specific changes in the CSF [14], or an apparently normal CT scan of the brain, which has been shown to be of lower sensitivity in detecting pituitary apoplexy compared to MRI scanning [15].

## Conclusion

Anticoagulation is well known as a precipitating factor for pituitary apoplexy. Like our patient, one case study has reported a patient with pituitary apoplexy precipitated by aspirin, clopidogrel and enoxaparin, although in that case the patient did not have a previously known pituitary adenoma [16]. Our case therefore highlights some important practice points: in patients who are already known to have a pituitary adenoma, this condition should be considered a relative contraindication for anticoagulation. These patients should be warned about the potential risks of anticoagulation with respect to their pituitary adenoma. If these patients are anticoagulated, a high index of suspicion of pituitary apoplexy should guide the clinician if they fall acutely ill: early hydrocortisone replacement should be instituted.

## Competing interests

The author(s) declare that they have no competing interests.

## Authors' contributions

All authors participated in the care of the patient described. TMMT drafted the manuscript. CC, ECIH, NMM, KM critically revised the content of the manuscript. All authors have read and approved the final version of the manuscript.
